# Factors associated with spontaneous abortion following intracytoplasmic sperm injection (ICSI)

**DOI:** 10.5935/1518-0557.20190028

**Published:** 2019

**Authors:** Zahra Basirat, Mehdi Kashifard, Masoumeh Golsorkhtabaramiri, Parvaneh Mirabi

**Affiliations:** 1 Infertility and Reproductive Health Research Center, Health Research Institute, Babol University of Medical Sciences, Babol, Iran; 2 Student Research Committee, Babol University of Medical Sciences, Babol, Iran

**Keywords:** miscarriage, galactorrhea, intracytoplasmic sperm injection, pregnancy

## Abstract

**Objective:**

The aim of this study was to describe the miscarriage rates and the factors associated with cases of spontaneous abortion observed in women offered intracytoplasmic sperm injection (ICSI).

**Methods:**

This cross-sectional study enrolled women who became pregnant with the aid of ICSI treated at the Babol Infertility Center (Iran) within a period of five years (2010-2015). Data were collected from patient charts and, in some cases, through phone calls. The study looked into the incidence of spontaneous abortion in women offered ICSI and the factors associated with miscarriage. The chi-square test, Fisher's exact test, and the t-test were used to analyze the data.

**Results:**

From a total of 145 pregnant women, 120 were included in our study. The prevalence of miscarriage was 20%. Galactorrhea was significantly more present in patients who had miscarriages (25% *vs.* 9.37%, *p*=0.04). There was a marked difference in the duration of infertility of miscarriage and non-miscarriage patients offered ICSI (6.6±8.3 *vs.* 4.9±7.3 years, *p*=0.05). No association was found between maternal age, BMI, cause of infertility, hormonal pattern, type of infertility, history of surgery, polycystic ovary syndrome, number of oocytes, or day of retrieval with miscarriage.

## INTRODUCTION

The acceptance of assisted reproductive technology (ART) treatments has grown among infertile couples attempting to achieve pregnancy (Jie *et al*., 2015). In the US, the use of intracytoplasmic sperm injection (ICSI) in fresh IVF cycles grew from 36.4% in 1996 to 76.2% in 2012 (Boulet *et al*., 2015). The International Committee for Monitoring Assisted Reproductive Technologies in Europe reported that ICSI is used in 65% of ART cycles (Dyer *et al*., 2016). ICSI patients usually undergo a higher number of IVF cycles, which could mean that ICSI is offered to patients with poorer prognoses (Tannus *et al*., 2017).

Pregnancies arising from ICSI have inherent risks not observed in pregnancies from normal conception. Patients offered ICSI are generally older than individuals attempting pregnancy through normal conception, and this difference may increase the risk of genetic disorders in the embryonic karyotype (Pendina *et al*., 2014). In addition, ICSI requires ovarian stimulation, which may increase the risk of multiple pregnancy, ectopic pregnancy, fetal growth restriction, and miscarriage (Zhu *et al*., 2016; Jackson *et al*., 2015; Egbe *et al*., 2016; Johnston *et al*., 2015; Perkins *et al*., 2015). The incidence of early pregnancy loss in ICSI patients undergoing fresh embryo transfers amounted to 14.9% *vs.* 26.2% in individuals submitted to frozen embryo transfers (Xu *et al*., 2017). Early miscarriages in patients given ICSI have decreased the success rate of ICSI. Several factors associated with spontaneous abortion in patients offered ICSI have been discussed. Most studies showed an association between maternal age and the BMI affecting oocyte or endometrial quality (Hahn *et al*., 2014; Metwally *et al*., 2010; Hourvitz *et al*., 2006). Other predictors have been related to the quality of the embryo, sperm, and IVF laboratory technology (Brandes *et al*., 2011; Xu *et al*., 2017). A few studies have also shown that fertility treatment itself may be a risk factor for early miscarriage. The risk of miscarriage was reportedly higher following frozen embryo transfers when compared to cases of spontaneous pregnancy (Xu *et al*., 2017).

According to previous studies, and considering the emotional and psychological burden imposed by miscarriage on infertile women offered ICSI, awareness of the risk factors associated with ICSI is of great help. Therefore, these findings may be used to evaluate assisted reproductive treatments and counsel infertile women suffering with miscarriage. This study aimed to determine the prevalence and the risk factors linked to miscarriage in women treated with ICSI.

## MATERIALS AND METHOD

This cross-sectional study included all infertile pregnant women treated with ICSI at the Fatemezahra Infertility and Reproductive Center within a period of five years (October 1, 2010 to September 30, 2015). The study protocol received approval from the Ethics Committee of the Bobal University of Medical Science (No. 697). All participants gave informed consent before joining the study.

The following inclusion criteria were adopted: patients on ICSI cycles aged 20-43 years receiving fresh embryo transfers. The included patients underwent biochemical and hormonal testing, in addition to vaginal ultrasound, hysterosalpingography (HSG), and semen analysis. HSG was performed before the patients were included in the study. Patients with structural anomalies underwent hysteroscopy to have the defects removed. Individuals with a history of recurrent miscarriage and uterine fibroids, patients with non-removable structural uterine anomalies, and women given frozen embryo transfers were excluded. Pregnant patients treated with the routine protocol in effect at our center (long protocol) were enrolled in the study. Patients whose charts missed information on pregnancy outcome were contacted by phone and interviewed to capture the missing data point.

Pregnancy was defined as follows: ß-HCG> 25u 16 days after the embryo transfer; patients with low ß-HCG levels on day 16 were tested again two days later. After pregnancy was confirmed, the patients were followed for ß-HCG levels and vaginal ultrasound examination was performed to detect a fetal heartbeat. Considering the patients included in this study, the risk factors collected and identified through questionnaires were maternal age, paternal age, the body mass index (BMI), polycystic ovary syndrome (PCOS), infertility causes, response to ovulation induction treatment including the number of oocytes, type of treatment type, hormone levels including follicle-stimulating hormone (FSH), luteinizing hormone (LH), thyroid-stimulating hormone (TSH), prolactin (PRL), on the third day of the menstrual cycle.

The patients were first categorized as follows based on age and BMI:

-Age: ≤30 years / 30.1-35 years / 35.1-40 years / >40 years 

-BMI: <18.5 (skinny), 18.5-25 (normal), 25.1-30 (overweight), 30.1-35 (obese), >35.1 (very obese)

They were subsequently divided into two groups: patients whose pregnancies ended in miscarriage and patients whose pregnancies produced live births. The chi-square test, Fisher's exact test and the t-test were used in statistical analysis. A *p*<0.05 was considered significant. All data were treated as confidential and used solely for the purposes of the study. No interventions were carried out during the study so as to minimize patient risk. Permission to access patient data was granted by the managers and staff in charge.

## RESULTS

The study included 860 patients, and 161 of them were pregnant. Of the 161 charts examined, 145 met the inclusion criteria. Patients with inaccurate or missing documents were also excluded. In the end, 120 cases were included in the study.

The pregnancy success rate was 18.72% (161 pregnancies in 860 patients), while the miscarriage rate was 20% (24 miscarriages in 120 cases). Of the 96 live births, 79 (82.3%) were from singleton pregnancies - 45 males (37.5%) and 34 females (35.4%); 15 (15.6%) were from twin pregnancies; and two (1.2%) were from triplet pregnancies.

The miscarriage rate was 40% in the galactorrhea group and 18% in the non-galactorrhea group. The difference between the two groups was statistically significant (*p*=0.04). However, prolactin levels did not show a significant relationship in either of the two groups (Table 1). The calculated effect size was -0.3.

**Table 1:** Comparison of hormone levels in women following ICSI

Hormone levels	Miscarriage group (%)	Live birth group (%)	*p*-value
Testosterone (nmol/L)	2.4±2.23	2.9±1.97	0.69
FSH (mlU/ml)	3.8±7.59	3.2±7	0.5
LH (mlU/ml)	2.5±5.6	3.3±5.8	0.84
PRL (mlU/l)	271.6±185.6	216.6±106.6	0.13
TSH (mlU/ml)	3.7±3	1.3±2.1	0.08

The mean age of the pregnant women enrolled in the study was 32.8±5.1, which was within the range of 20 to 43, while the mean age of their partners was 32.8±6.0. The mean duration of infertility was 5.26±3.8 years, which was within the range of one to 19 years. No association was found between maternal age and miscarriage rate in the age groups (*p*=0.69). In the other age groups - individuals aged >35 and ≤35 years, with respective miscarriage rates of 27.3% and 19.3% - the difference was not significant (*p*=0.69). The calculated effect size showed that the chances of success decreased by 8% among individuals aged >35 years (Effect size =- 0.1). The age groups of the pregnant women included in the study following ICSI are shown in Table 2.

**Table 2:** Age groups of pregnant women following ICSI

Age	Miscarriage group (%)	Live birth group (%)	*p*-value
≤30	16 (18)	73 (82)	0.69
30.1-35	5 (25)	15 (75)	
35.1-40	1 (11.1)	8 (88.9)	
>40	2 (100)	0	

The mean BMI of the miscarriage group was 26±3.7, *versus* 26.1±3.8 of the non-miscarriage group, suggesting the absence of a significant correlation between the BMI and pregnancy rate (*p*=0.9). Table 3 shows the stratification of the BMI for pregnant women following ICSI.

**Table 3:** BMI groups of pregnant women following ICSI

BMI	Miscarriage group (%)	Live birth group (%)	*p*-value
≤18.5	0	1 (100)	0.63
18.5-25	14 (23.3)	46 (76.7)	
25.1-30	8 (16.7)	40 (83.3)	
30.1-35	1 (11.1)	8 (88.9)	
>35.1	1 (50)	1 (50)	

Male factor infertility was diagnosed in 31.7% (38 patients) of the cases, while female factor was observed in 12.5% (15 patients) of the cases. Fifty-five percent (66 patients) had both types of infertility, and 8% (one patient) had unexplained infertility. No association was found between cause of infertility and miscarriage (*p*=0.55) (Table 4).

**Table 4:** Causes of infertility in pregnant women following ICSI

Cause of Infertility	Miscarriage group (%)	Live birth group (%)	*p*-value
Male factor	5 (13.2)	33 (86.8)	0.63
Female factor	3 (20)	12 (80)	
Male & female factor	16 (24.2)	50 (75.8)	
Unexplained	0	1 (100)	

The mean duration of infertility in the miscarriage group was 6.6±3.8 *vs.* 4.9±3.7 years in the non-miscarriage group, indicating the existence of a significant relationship between duration of infertility and miscarriage rate (*p*=0.05). Primary infertility was observed in 77.5% (93 patients) of the cases and secondary infertility in 22.5% (27 patients) of the cases. The miscarriage rates in the above groups were 19.4% and 22.2%, respectively. Statistically, there was no significant relationship between the type of infertility and miscarriage (*p*=0.73). The miscarriage rate was 23.8% in patients with PCOS *vs.* 17.9% in patients without PCOS. The miscarriage rate was higher in the group with PCOS, but the difference was not statistically significant (*p*=0.44).

The number of ampules used in ovulation induction, the day of oocyte retrieval, the number of eggs, and endometrial thickness were not significantly correlated with miscarriage (Table 5).

**Table 5:** Mean values of related parameters seen in women following ICSI

Parameter	Miscarriage group (Mean±SD)	Live birth group (Mean±SD)	*p*-value
Number of ampules of HMG used	10.4±8.8	6.6±7.3	0.39
Number of ampules of GONAL used	11.1±21.7	11.2±20.17	0.56
Number of ampules of FOSTIMON used	8.4±2.8	7.4±2.7	0.91
Day of oocyte retrieval	2.1±15.2	1.2±15.3	0.72
Number of oocytes	7±11	5.8±10.8	0.93
Endometrial thickness	1.2±10.1	1.6±10.3	0.71

As far as having a history of surgery is concerned, 26.7% (32 patients) of the cases had undergone surgery including Cesarean sections, laparoscopy/laparotomy, appendectomy, D & C (dilation and curettage), procedures for the removal of ovarian or breast cysts, and endometriomas. No association was found between having a history of surgery and miscarriage (*p*=0.4).

Sixty-two (84.9%) of the 73 patients who underwent hysterosalpingography had normal HSG findings. Eleven (15.1%) had abnormal HSG findings and suffered from conditions such as endometriosis, tubal obstruction, and hydrosalpinx. However, no significant correlation was found between the two groups in terms of miscarriage rates (*p*=0.3).

## DISCUSSION

According to our results, longer duration of infertility and galactorrhea were associated with increased miscarriage rates, although miscarriage patients did not have higher serum prolactin levels. If we consider that the related *p*-value presents only the chance responsible for the observed difference in the women with galactorrhea, the calculated effect size may present an assurance that there is an association between galactorrhea and miscarriage rates. In woman with galactorrhea, the probability of having a successful pregnancy decreases by 13.9%. Each 7.2 women with galactorrhea have at least one miscarriage when compared to women without galactorrhea (number needed to harm).

Galactorrhea is a relatively common problem. However, it is often missed at presentation. Ugwa *et al*. (2016) reported that most of their patients had normal prolactin levels regardless of galactorrhea. A possible implication is that routine breast examination might be needed for infertile women throughout infertility treatment to allow earlier diagnosis of galactorrhea.

The miscarriage rate in our study was 20%. The miscarriage rate reported for patients submitted to ART was 21% in a study by Wang *et al*. (2001) and 18% in a study by Aflatoonian *et al*. (2011). The above rates are in agreement with our findings. The causes for the high miscarriage rates seen in these treatment methods included older maternal age, history of miscarriage, the procedure used for ART, increased genetic problems inherent to these methods, and the causes of infertility, infertility itself, multiple pregnancies, embryo quality, hyperstimulation, high and low BMI levels, and increased risk of miscarriage.

Although female age and BMI are first line prognostic factors in human reproduction, we did not find significant differences between the age or BMI groups with miscarriage. Winter *et al.* (2002) concluded that there was no significant relationship between age, obesity, and the miscarriage rate, while Dai*et al*. (2018) pointed out that women over the age of 40 had significantly higher early miscarriages rates (60.6%) than women under the age of 40. Weight gain and obesity had no effect on miscarriage rates in a study by Tian *et al*. (2007). However, Moragianni *et al*. (2012) concluded that ART in patients with a BMI ≥ 30 yielded significantly lower implantation and clinical pregnancy rates. In our study, the non-significant association can be attributed to the lower maternal age and BMI. Most of the patients in our study were younger than 30 (74.2%), and 50% of the women had a normal BMI within the range of 18.5-25, and 40% were obese (BMI 25.1-30). This finding may have been affected by the limited size of our sample. Our results, therefore, must be interpreted with caution. Calculating the effect size revealed a weak relationship between age >35 and miscarriage rate, and that the chances of having a successful pregnancy dropped by 8% in the group aged >35 years. We also calculated the effect size for the association between BMI >30 and miscarriage, and found the two were poorly associated and the chances of having a successful pregnancy dropped by 72.3% in women with a BMI >30. Further studies with more focus on age subgroups and their associations with miscarriage are needed. A marked association was found between duration of infertility and rate of miscarriage. This finding may be attributed to possible increases in age or BMI among couples with the longer duration of infertility.

The miscarriage rate was higher in the PCOS group, but the difference was not statistically significant. Although Beydoun *et al*. (2009) reached the same conclusion, Kamalanathan *et al*. (2013) and Luo *et al.* (2017) found that the rate of miscarriage in PCOS patients was higher than in non-PCOS patients, and that the elevated prevalence of miscarriage was related to a high prevalence of obesity in individuals with PCOS. In our study, more than half of the participants had a normal BMI, which to some extent precluded the addition of the effects of a higher BMI and having PCOS.

The results showed that there was no significant correlation between rate of miscarriage and cause of infertility. Bahceci & Ulug (2004) found no significant association between cause of infertility and rate of early miscarriage. This is not consistent with the results described by Hajishafiha *et al*. (2011), in a study that found a significant relationship between the causes of infertility and the rate of miscarriage.

## CONCLUSION

Increasing galactorrhea and the duration of infertility may increase the risk of miscarriage. Therefore, women require early care and counseling before pregnancy along with meticulous care during the pregnancy. In doing so, it might be possible to minimize the adverse effects of pregnancy for patients.
